# An untapped resource? Opportunities for faculty-librarian collaboration to enhance drug information resource utilization in pharmacy education

**DOI:** 10.5195/jmla.2022.1486

**Published:** 2022-10-01

**Authors:** Kayce D. Gill, Robin Parker

**Affiliations:** 1 kayce.gill@vanderbilt.edu, Health Sciences Collections Librarian, Annette and Irwin Eskind Family Biomedical Library and Learning Center, Vanderbilt University, Nashville, TN.; 2 robin.parker@lipscomb.edu, Assistant Professor of Pharmacy Practice, Department of Pharmacy Practice, Lipscomb University College of Pharmacy, Nashville, TN.

**Keywords:** Pharmacy education, student pharmacists, drug information, Information literacy, Instruction, Interprofessional collaboration, health sciences librarians

## Abstract

**Background:**

Doctor of pharmacy educational accreditation standards state student pharmacists should be able to evaluate the scientific literature as well as critically analyze and apply information in answering drug information questions. Student pharmacists often struggle with identifying and using appropriate resources to answer medication-related questions. To ensure educational needs were met, a college of pharmacy hired a health sciences librarian to support the faculty and students.

**Case Presentation:**

The health sciences librarian collaborated with faculty and students throughout the doctor of pharmacy curriculum to identify and address any gaps related to appropriate drug resource utilization. Adding instruction time to the new student pharmacist orientation, coursework throughout the first year of the pharmacy program, and a two-semester evidence-based seminar course provided opportunities for the health sciences librarian to work with student pharmacists in the areas of library resource access, instruction on drug information resources, and evaluation of drug information found on the internet.

**Conclusion:**

The deliberate inclusion of a health sciences librarian into the doctor of pharmacy curriculum can benefit faculty and students. Opportunities for collaboration are available throughout the curriculum, such as providing instruction for database utilization and supporting the research activities of both faculty and student pharmacists.

## BACKGROUND

When pursuing a doctorate of pharmacy, student pharmacists need to master the essential skills of quickly and efficiently retrieving and evaluating information to apply to patient care. The Accreditation Council for Pharmacy Education affirms this need in Standard 1 of the 2016 Standards citing that student pharmacists should be able to evaluate the scientific literature as well as critically analyze and apply information in answering drug information questions [1]. As the rate of newly created health-related information has continued to grow exponentially, colleges of pharmacy must find ways to help student pharmacists learn the skills necessary to navigate the rapidly changing information landscape.

One method to address this need is to incorporate a librarian with subject matter expertise into a pharmacy curriculum. Colleges of pharmacy and libraries have a long history of collaborating to ensure student pharmacists learn drug information skills such as finding primary literature, searching drug information resources, and citing in AMA style [2-8]. The few studies that focus on the integration of drug information skills and librarian involvement throughout the didactic pharmacy curriculum [3, 4, 9] are more than fifteen years old. More recent studies report on incorporating a librarian into a specific course or rotation [10, 11] or using gamification techniques as active learning strategies [8,12]. The study most similar to the present study, Wu et al. [13], reported the experience of becoming an embedded librarian in a college of pharmacy and used a variety of methods to track changes pre- and post-embedding, such as course contact hours and the number of reference questions asked. However, reported outcomes were limited to increased resource utilization without additional information regarding specific learning activities used by the librarian once embedded into the pharmacy program. This case presentation examines scaffolded librarian involvement throughout a college of pharmacy's curriculum and provides examples of how librarians can become involved.

In 2019, Lipscomb University's College of Pharmacy and Health Sciences (COPHS) hired a health sciences librarian as part of the college faculty to work with faculty, staff, and students in the health sciences programs. The COPHS is comprised of programs in cardiovascular perfusion, dietetics, kinesiology, nursing, pharmacy, and physician assistant studies; however, pharmacy faculty expressed the greatest need for library support during department meetings, email requests, and office conversations. Previously, the pharmacy faculty had access to a liaison librarian provided by the university library, but the liaison responsibilities were limited to communication between the college and library, processing annual resource renewals, and maintaining the pharmacy research guide. The new health sciences librarian position was responsible for collection development, information services, instruction, and research support for the health sciences programs. Though the position reported to the Vice Provost of Health Affairs, the health sciences librarian worked with the university library to ensure all health sciences resources were cataloged and available in the discovery system and databases list.

During department meetings and individual meetings, several pharmacy faculty members and preceptors expressed concern about student pharmacists' drug information skills; specifically, the ability to identify and use appropriate drug information resources to answer drug information questions, the ability to effectively find and evaluate primary literature, and the ability to quickly access the library's resources. This paper describes how faculty in a college of pharmacy collaborated with a health sciences librarian to alleviate the faculty-identified problems and improve the student learning experience. See Table 1 for a complete list of the pharmacy courses that utilized embedded librarian expertise as well as the topics and related learning activities covered in the sessions. Selected courses are discussed in the context of the identified issues in the case presentation that follows. Planning for these sessions started in spring and summer of 2019 and were fully implemented during the 2019-2020 academic year.

**Table 1:** Librarian Involvement in Pharmacy Curriculum (Average class size 60-65).

Course	Year in Curriculum	Contact Hours	Topic	Related Learning Activities
Pharmacy Orientation	P1 (Fall)	0.5	Introduction to library resources & services	Register for MyAccess & APhA personal accounts
Foundations in Pharmacy Practice	P1 (Fall)	2	Introduction to drug information resources (Lexicomp, Micromedex, Natural Medicines), PubMed, & AMA style	Demos of resources & virtual escape room game
Introductory Pharmacy Practice Experience (IPPE) Simulation	P1 (Fall)	0.5 weekly x 15 weeks	Weekly drug information questions	Discussion and feedback on students' answers
Biostatistics & Medical Literature Evaluation	P1 (Spring)	4	Advanced training on drug information resources (Lexicomp, Micromedex), PubMed, & AMA style	Demos of resources & virtual escape room game
Health Care Informatics	P2 (Fall)	2	Drug information resources (Epocrates, Lexicomp, Micromedex) & connection to health care informatics	Demos of resources & competition style questions asked through Mentimeter
Natural Medicines	P2 (Fall)	3	Introduction to drug information resources for natural medicines, herbs, & supplements (Natural Medicines, National Center for Complementary Integrative Health, Office of Dietary Supplements, Medline Plus)	Demos of resources & virtual escape room game
Clinical Seminar I	P3 (Fall)	2	Searching for primary literature (original research) using PubMed	Demo of PubMed advanced searching techniques & in-class journal club worksheet
Pharmaceutical Sciences Academic Research Elective (Independent study with 1 student)	P3 (Fall)	1 weekly x 15 weeks	Introduction to systematic reviews & guidance on developing one	Weekly activities for the various steps of a systematic review
Applied Pharmacotherapy	P3 (Fall)	0.5	Searching for clinical practice guidelines using ECRI Guidelines Trust and PubMed	Demo of resources & account registration
Clinical Seminar II	P3 (Spring)	2	Developing a research topic, finding supporting evidence, & plagiarism	In-class worksheet on developing a topic & finding supporting evidence
Pharmacotherapy VI	P3 (Spring)	1	Refresher on drug information resources (Lexicomp, Micromedex) & PubMed before Advanced Pharmacy Practice Experience (APPE) start	Q&A session with demos by request

## CASE PRESENTATION

### Drug Information Resource Utilization

In the age of widespread internet use and comfort with search engines such as Google, faculty noticed an overwhelming issue of student pharmacists using inappropriate resources to answer medication-related questions. Student pharmacists often chose the first linked result, which many times included direct-to-consumer advertisements or unreliable internet websites. Despite COPHS having access to numerous reputable drug information databases, student pharmacists' comfort with locating and utilizing such resources as Lexicomp and Micromedex was deficient due to a lack of formalized training on using these resources.

To address this gap, beginning in Fall 2019, the health sciences librarian added a thirty-minute session to the new student pharmacists' orientation. During the orientation, the health sciences librarian focused on introducing basic library information: library location, hours, the health sciences librarian's contact information and office location, and the pharmacy research guide. The health sciences librarian also demonstrated how to use AccessPharmacy and APhA PharmacyLibrary, which provide many of the program's required textbooks. This time was also spent helping student pharmacists create their personal APhA and MyAccess accounts enabling them to save textbooks to a centralized location, use the study tools to reinforce learning, and track progress on practice problems.

Pharmacy practice faculty identified the first-year course Foundations in Pharmacy Practice as a place for intentional drug information instruction. A two-hour drug information session was previously taught by pharmacy practice faculty several weeks into the semester. The health sciences librarian assumed responsibility for this session and moved it to the first week of the semester to lay the foundation for student pharmacists to build upon as they progressed through their coursework. The health sciences librarian taught the two-hour drug information lecture for the first time in the Fall 2019 and decided to not make any changes to the content, except for removing AccessPharmacy and APhA PharmacyLibrary, as those resources were covered during orientation. This decision provided the health sciences librarian a baseline understanding of the student pharmacists' introduction to drug information resources, which could be used to revise the content for the following years. Reflecting on the experience, the health sciences librarian realized that student pharmacists were overwhelmed by the amount of information shared in the two-hour session, and most of them performed poorly on the related homework assignment. To address this information overload, the health sciences librarian collaborated with pharmacy faculty members to identify the introductory drug information skills that first-year student pharmacists should know by the end of the first semester. The librarian used that information to redesign the content delivery for the fall of 2020 into an interactive lecture that introduced the drug information resources (Lexicomp, Micromedex, and Natural Medicines) and highlighted the important elements for first-year student pharmacists to master, such as looking up a medication, navigating through a drug monograph, and searching for a drug by indication. The health sciences librarian used an audience response tool (Mentimeter) for active engagement during the lecture and designed a virtual escape room game to help student pharmacists practice navigating the various resources and apply information gained from searching databases such as Lexicomp, Micromedex, Natural Medicines, and PubMed. In a voluntary survey offered at the end of the session, several student pharmacists felt that the exercise was a meaningful experience in preparing them for patient care in the future.

**Figure 1:**
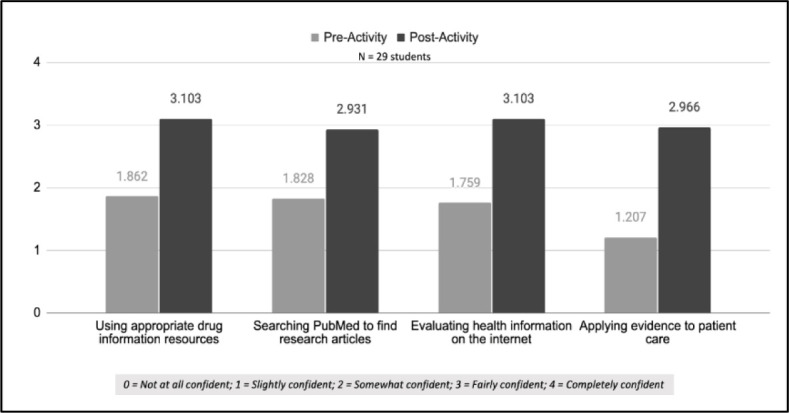
IPPE Drug Information Self-Efficacy Survey Results

To further reinforce using appropriate drug information resources, the health sciences librarian collaborated with pharmacy practice faculty to incorporate targeted drug information questions into a first-semester introductory pharmacy practice experience (IPPE) simulation course. This project began in Fall 2019 and continued in Fall 2020 and Fall 2021. Each week, student pharmacists were given three drug information questions and instructed on which specific resources to use to answer the questions. Student work was submitted through a Google Form outside of class time. Question type and resource utilization built in complexity each week. The health sciences librarian and a pharmacy practice faculty member provided feedback on the student pharmacists' answers, highlighting commonly missed concepts. During the feedback sessions, the faculty member and health sciences librarian demonstrated their search strategies to find the necessary information to answer the drug information questions, showcasing the various approaches that can be taken to best answer a drug information question, such as MeSH searching versus keyword searching to address drug information questions using PubMed. Demonstrating different ways to search for drug information also emphasized the importance of evaluating the information and its source. During this course in Fall 2021, student pharmacists took an optional, non-incentivized pre-activity and post-activity survey on their confidence and abilities using the resources to answer drug information questions. In a Lipscomb University institutional review board-approved study, first-year students (29 out of 34 completed all questions and were included in the analysis) were asked to rate their confidence using appropriate references to answer medication-related questions, searching PubMed, evaluating health information on the internet, and applying medication information to patient care. At the end of the IPPE simulation course, student pharmacists reported a statistically significant increase in their confidence in using the drug information resources (P<0.05 for all questions; See Figure 1; full survey instrument available in supplementary materials available at https://osf.io/cr967/)

Student pharmacist skills were assessed using 2 case-based questions about selecting the appropriate resource to answer the drug information questions. The first question asked students to select the most appropriate resource for answering a question about heartburn treatment during pregnancy. At the end of the IPPE simulation course, student pharmacists' ability to select the appropriate drug information resource improved from 86% (pre-activity) to 93% (post-activity). The second question asked students to select the most appropriate resource for finding evidence about efficacy and safety comparing two medications, intending for students to grasp that locating a randomized clinical trial through PubMed would be an appropriate result; however, the selection of PubMed as the appropriate resource only increased from 24% to 38%. The low selection of PubMed as an appropriate resource highlights the continued need to incorporate additional instruction on the role of PubMed in answering drug information questions.

### Finding and Evaluating Primary Literature

The critical appraisal of medical literature is an increasing area of focus and opportunity for student pharmacists and graduates. To address this need, a two-semester course in the third year on locating, evaluating, and presenting evidence-based pharmacy practice information was identified as a course to collaboratively redesign with the help of the health sciences librarian. In pharmacy education, evidence-based information resources are described as primary (research articles), secondary (databases like PubMed), and tertiary (textbooks and drug information resources). The first-semester presentation is a critique of an original research article, and the second-semester presentation is a comprehensive topic update that requires student pharmacists to identify and evaluate three original research articles. The course structure supported student pharmacists' comfort with presenting; however, it failed to provide meaningful opportunity to find and evaluate medical literature and identification of its pertinence to current scientific evidence.

To better support student learning, in the first semester, the health sciences librarian added a 15-minute demonstration on citation searching using PubMed and accessing full text articles using the browser extension, LibKey Nomad, to aid students, who are given a citation and expected to locate the full text articles. Information about using interlibrary loan was also shared in case an article was unavailable through the library's resources. The health sciences librarian also designed a two-hour lecture on developing a search strategy, finding background information in tertiary sources, and searching PubMed for additional evidence. The course coordinator and health sciences librarian revised a college-specific journal club outline to help student pharmacists identify key elements of a research article (Worksheet available at link in data availability statement). Using a scaffolded approach to learning, student pharmacists were then able to use the completed worksheet as a guide for developing their presentation slides for the evidence-based pharmacy practice course presentation.

For the second semester, student pharmacists were responsible for researching a topic that is timely to patient care and the practice of pharmacy and preparing an update deemed appropriate for continuing education requirements. The health sciences librarian developed an interactive two-hour lecture on developing a research topic and utilizing PICO to develop keywords and an expanded PubMed search strategy, reinforcing the previous semester's instruction on PubMed searching. The health sciences librarian also created an in-class worksheet that required students to describe their topics, create a PICO to identify important aspects of their topics, develop keywords, identify Medical Subject Headings (MeSH) vocabulary, search secondary sources (PubMed) for primary literature (research articles), and cite their sources in AMA style. The worksheet provided a framework for independent presentation development with an evidence-based foundation. Faculty have expressed that topic, evidence, and presentation quality increased after librarian expertise and instruction were incorporated into the course structure.

### Access to Library Resources

**Figure 2:**
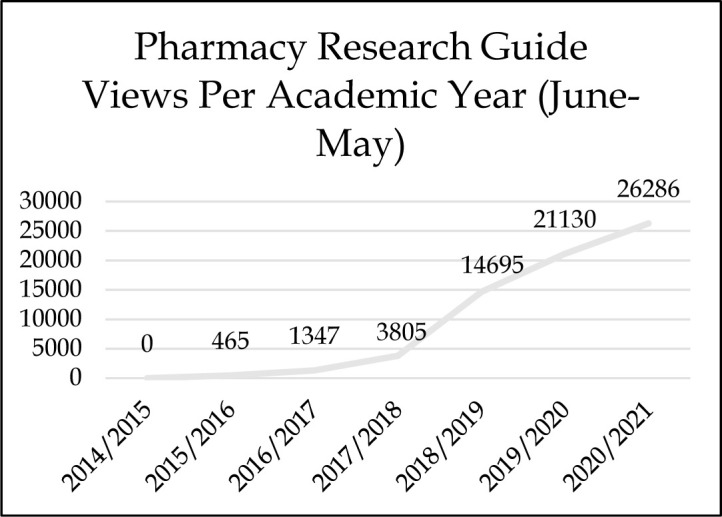
Pharmacy Research Guide Views Per Academic Year

A major issue that plagued pharmacy faculty and student pharmacists was inconsistent and unreliable access to pharmacy-related electronic resources because they were set up with unique usernames and passwords instead of IP address authentication. The health sciences librarian streamlined all electronic resources to IP address authentication and moved them to the university's single sign-on solution, which has resulted in fewer reported access issues. Additionally, the health sciences librarian worked with pharmacy faculty to build a new pharmacy-focused research guide that met the needs of the student pharmacists and faculty. The pharmacy research guide provides a single website for quick access to all pharmacy-related electronic resources as well as ways to email and schedule research appointments with the health sciences librarian. A pharmacy research guide was originally created in 2014 using LibGuides, but the guide was not marketed well and had low usage. The guide was revised in 2017, and the new guide designed with input from faculty and students was released in February 2019 and added to the COPHS website, with a resulting increase in utilization demonstrated in *Figure 2 Pharmacy Research Guide Views Per Academic Year.* The health sciences librarian introduces the pharmacy research guide to new student pharmacists during the fall orientation to establish the importance of this webpage as the centralized location for pharmacy-related library resources.

## DISCUSSION

Drug information skills are a vital part of student pharmacists' education, and as demonstrated by this case study, pharmacy faculty and health sciences librarians can collaborate to ensure student pharmacists receive the training they need. The experience of integrating information literacy touchpoints throughout a pharmacy curriculum was a rewarding experience for both faculty and student pharmacists. Faculty collaboration was an essential part of making this a successful process. One key step in this process was a mandatory pharmacy department meeting where the health sciences librarian was introduced as a fellow faculty member and collaborator. During that meeting, the health sciences librarian reviewed the existing resources with the faculty and asked for suggestions for new resources as well as identified issues faculty and students were experiencing. Attending regular department meetings kept the health sciences librarian aware of important changes in the college but also helped reinforce the idea of the health sciences librarian as part of the team.

The health sciences librarian discovered that one of the best methods for connecting with faculty was scheduling one-on-one meetings to discuss how they were currently using library resources in their courses, deficiencies they identified in student pharmacists, and skills they wanted student pharmacists to master before graduation. To effectively establish a librarian-teaching faculty collaboration required the health sciences librarian to gain an understanding of the program's curriculum and learning objectives. Through this process, the librarian identified courses where collaboration would likely be welcomed. Approaching the teaching faculty and highlighting the skillset strengths of librarians clarified the scope and depth of educational opportunities for students. Teaching faculty were eager to partner with the health sciences librarian once the librarian demonstrated the skillsets inherent to librarian expertise, such as supporting a systematic approach to research and expert searching.

Perhaps the best opportunity to start supporting student learning was speaking at the program's orientation, as this proved to be an effective strategy to engage students and ensure they recognize the support the librarian can provide throughout their education. As demonstrated through the successes of the various learning activities mentioned throughout this case report, engaging with a health sciences librarian enhanced student pharmacists' comfort and ability utilizing appropriate resources. Anecdotal data from conversations with first-year student pharmacists identified the escape room game in the Foundations in Pharmacy Practice and the feedback sessions in the IPPE Simulation course as the most beneficial. Student pharmacists felt that the escape room game provided a low-stakes environment to learn key aspects of the drug information tools without the added stress of finding a correct answer for a grade. Student pharmacists also identified the demonstration of various searching methods during the IPPE Simulation course feedback as beneficial due to the faculty member's and librarian's different search techniques. In hallway conversations, several third-year student pharmacists commented that the worksheets in Clinical Seminar I and II greatly helped them organize their article critiques and develop their presentation slides. The incorporation of librarian expertise has provided the opportunity for meaningful improvement in student pharmacists' drug information skills and has strengthened the pharmacy faculty members' perception of the importance of collaborating with librarians in the future.

## LIMITATIONS

A limitation of this study is that the university's College of Pharmacy and Health Sciences hired a dedicated health sciences librarian, whose responsibility was to support *only* that college's faculty, staff, and students. Since the health sciences librarian was not part of the university library system and did not attend library staff meetings, work the library reference desk, participate in the library's instruction program or other library activities, the librarian was able to dedicate time to developing relationships with the various health sciences programs and faculty through departmental meetings, one-on-one consultations, and committee service as well as working on curriculum development. The authors acknowledge that having a dedicated librarian as part of college and not a library is a unique situation and a luxury that most libraries cannot afford. Despite that, some of the authors' recommendations still hold true to building effective and lasting partnerships. For example, finding the time to attend an occasional departmental meeting ensures that faculty and staff remember that the library exists and has valuable resources and can help establish the library as a partner in curriculum development.

## Data Availability

Example drug information questions from the IPPE simulation course, full survey and complete results from the IPPE Drug Information Survey, worksheets used in the third-year course, and the virtual escape room game for Foundations in Pharmacy Practice are available at https://osf.io/cr967/.

## SUPPLEMENTAL FILES

**Full survey instrument available in supplementary materials available at** https://osf.io/cr967/.
